# Tunable Room Temperature THz Sources Based on Nonlinear Mixing in a Hybrid Optical and THz Micro-Ring Resonator

**DOI:** 10.1038/srep09422

**Published:** 2015-03-24

**Authors:** Raju Sinha, Mustafa Karabiyik, Chowdhury Al-Amin, Phani K. Vabbina, Durdu Ö. Güney, Nezih Pala

**Affiliations:** 1 Department of Electrical and Computer Engineering, Florida International University, 10555 W Flagler St., Miami, FL. 33174, USA; 2 Department of Electrical and Computer Engineering, Michigan Technological University, 1400 Townsend Dr., Houghton, MI. 49931, USA

## Abstract

We propose and systematically investigate a novel tunable, compact room temperature terahertz (THz) source based on difference frequency generation in a hybrid optical and THz micro-ring resonator. We describe detailed design steps of the source capable of generating THz wave in 0.5–10 THz with a tunability resolution of 0.05 THz by using high second order optical susceptibility (χ^(2)^) in crystals and polymers. In order to enhance THz generation compared to bulk nonlinear material, we employ a nonlinear optical micro-ring resonator with high-Q resonant modes for infrared input waves. Another ring oscillator with the same outer radius underneath the nonlinear ring with an insulation of SiO_2_ layer supports the generated THz with resonant modes and out-couples them into a THz waveguide. The phase matching condition is satisfied by engineering both the optical and THz resonators with appropriate effective indices. We analytically estimate THz output power of the device by using practical values of susceptibility in available crystals and polymers. The proposed source can enable tunable, compact THz emitters, on-chip integrated spectrometers, inspire a broader use of THz sources and motivate many important potential THz applications in different fields.

The Terahertz (10^12^ Hz) region of electromagnetic spectrum covers the frequency range from roughly 300 GHz to 10 THz, which is in between the microwave and infrared regimes. This THz regime has potential applications in medical imaging, security screening, remote sensing, chemical detection, quality control of semiconductor devices, space research and tactical imaging^1^,^2^,^3^,^4^,^5^,^6^,^7^. One significant impediment in this field is the cost and complexity associated with THz sources. The increasing interest in the development of novel THz sources has stimulated in-depth studies of microscopic mechanisms of THz field generation in conventional semiconductors, electro-optic materials, and an extensive search for new materials and devices to be employed in THz generation and detection. Among all the major approaches of developing the THz sources, the utilization of optical methods has been the major technique for the demonstration of many THz applications. This method includes photoconductive antenna, optical rectification, optical parametric oscillation and difference frequency generation (DFG)^8^,^9^,^10^,^11^. We summarize state-of-the art THz emitters in Table 1 with respect to spectral range, output power, operating temperature, tunability and size. Despite the tremendous research and development efforts, all the available THz emitters suffer from either being large and complex or requiring high input power and being expensive or very low operating temperatures, lack of tunability and very low output power.

So far THz sources based on DFG are designed and realized mostly in bulk crystals^11^. Recently, intra-cavity DFG, cavity enhanced DFG, rib waveguides for DFG, triply resonant photonic resonators for DFG, and DFG in nonlinear polymer cladding have been reported for THz generation^12^,^13^,^14^,^15^,^16^. Current approaches to using χ^(2)^ nonlinearities suffer from low conversion efficiencies^16^. However, polymers with very high value of second order nonlinearity (r_33_ ~ 132 pm/V) are demonstrated, which became commercially available recently^17^,^18^. It is expected that with further improvement, r_33_ values approaching 500 pm/V, which is about one order of magnitude stronger than typical nonlinear crystals will be possible^16^. Moreover, efficiency of nonlinear processes can be significantly increased by using high-Q resonators, where photons make multiple round trips on resonance resulting in the optical intensity being enhanced by a factor of the finesse^19^,^20^. In this article, for the first time we propose and investigate in detail a tunable, compact THz emitter based on DFG using a hybrid optical and THz micro-ring resonator. We analytically estimate the THz output power of the proposed THz source device for some practical crystals and polymers and present detailed simulation results employing commercial FEM and FDTD simulation tools^21^,^22^.

## Results

In this section, we first describe the proposed device and working principles in detail. Following, the design process of the micro-ring resonators and phase matching are presented. Then simulation results of the source device carried out in commercial simulation tools are presented in detail. At the end, the expected output power of the proposed emitter is calculated analytically for selected nonlinear optical materials using the DFG theory.

### Proposed device

**Figure 1:**
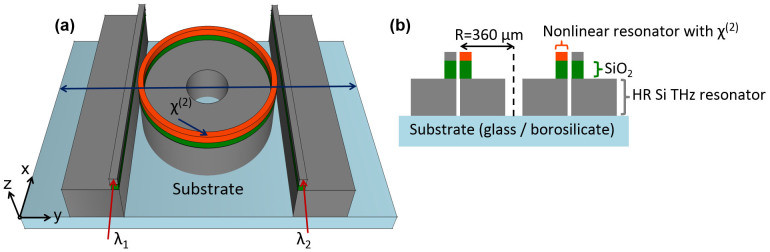
Proposed tunable THz source. (a) 3D schematic of the THz source device based on a hybrid nonlinear optical and THz micro-ring resonator. A pair of straight bus waveguides is employed to carry in the input infrared waves for DFG. To carry out the generated THz in the ring, another pair of straight THz waveguides is placed at close proximity to the THz ring resonator. (b) Cross-sectional schematic of the proposed source device.

Compactness, broad tunability, simple alignment, and stable THz output are sought after properties in the new generation THz sources. We propose a tunable, compact room temperature THz source that could radiate in 0.5–10 THz with a tunability resolution of 0.05 THz. Figure 1 shows the proposed hybrid tunable THz source device with a 3D schematic and cross-section. The hybrid device consists of an optical ring resonator (orange colored) with the outer radius of 360 μm, width of 0.6 μm, thickness of 0.5 μm for the investigated case and with a material (e.g. polymer) having second order nonlinear susceptibility (χ^(2)^). We employ a pair of optical straight bus waveguides placed at two opposite sides of the nonlinear ring in order to carry in the appropriate input infrared pump and idler waves so that it could generate DFG in the desired THz regime. These input waves will couple via evanescent field waves to the nonlinear ring where they are enhanced due to high Q factor of the resonator. The enhanced input waves make multiple round trips in the ring with resonant optical modes and generate THz waves via DFG phenomenon while interacting with the nonlinear material. Since the nonlinear ring cannot sustain the generated THz waves within itself due to their longer wavelength compared to input infrared, a THz ring resonator made of high resistivity Si is added with the same outer radius of 360 μm, 200 μm width and 120 μm thickness underneath the nonlinear ring resonator. The generated THz waves propagate in the THz ring with resonant THz modes satisfying phase matching condition. Optical and THz resonators are separated by an insulation layer of 1 μm thick SiO_2_ ring with the same width as the optical ring so that optical waves could propagate and interact well with the nonlinear material in the optical ring without being depleted via evanescent coupling to the THz ring made from high-index Si. A pair of THz straight waveguides is placed underneath the input waveguides with the same SiO_2_ insulation layer so that it could out-couple the THz waves from the THz ring resonator and guide them to any point of interest including an antenna for out-coupling to free space. It should be noted that the two THz waveguides can be combined into one with appropriate design to guide the total THz power depending on the application. The device could be permanently bonded with quartz glass or borosilicate substrate. By keeping the idler input wave fixed at 1550 nm and varying the pump wave from 1546 nm to 1474 nm at each 0.4 nm interval while satisfying the resonance condition of the ring resonator, the proposed THz source could emit THz radiation in the 0.5–10 THz range with tunability resolution of 0.05 THz.

### Micro-ring resonator

Micro-ring resonator is now considered as one of the most important building block of integrated photonics and has gained widespread interest over the past few years. It consists of a waveguide in a closed loop, commonly in the shape of a ring or racetrack. When placing the loop within close proximity of an input waveguide, light can be coupled into the cavity via evanescent field and light waves can propagate to circulate around the periphery of the cavity. Resonance takes place because of the constructive interference for light whose phase change after each full trip around the closed loop is an integer multiple of 2π, i.e., in phase with the incoming light. Waves that do not meet this resonance condition are transmitted through the input waveguide. Resonance wavelength of the ring is defined by^23^
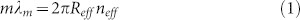
where *m*, *λ_m_*, *R_eff_*, and *n_eff_* are resonant azimuthal mode number, resonant wavelength with mode number *m*, effective radius of the ring and effective mode index of the material in the ring respectively. Minimum attenuation for the optical wave propagation in the ring resonator can be achieved if it is properly designed. One of the important parameters for designing a ring resonator is free spectral range (FSR), which is defined as the distance between two consecutive resonant peaks in the ring. The lower the FSR the higher the number of resonant absorption peaks in the ring for a particular bandwidth. The lower FSR is needed because tunability resolution of the source device is directly related to the number of resonant peaks in optical region. The relation between FSR and radius of the ring is defined by^23^
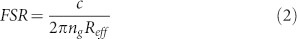
where *c* is the speed of light, *n_g_* is the group index and *R_eff_* is the effective radius of the ring. Group index takes into account the dispersion of the ring waveguide and is defined by^23^
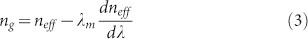


**Figure 2:**
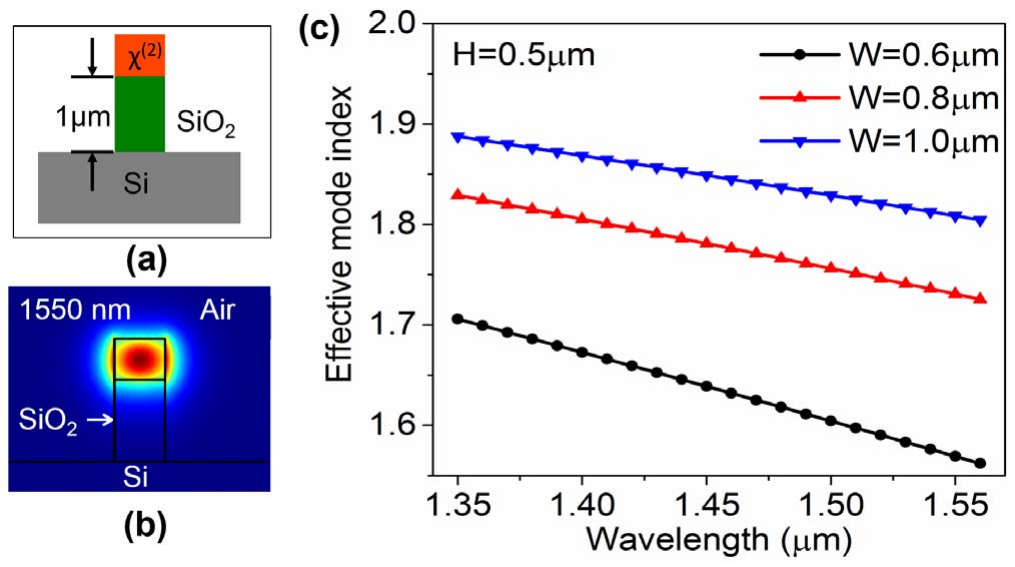
Dispersion of nonlinear optical waveguide. (a) Cross-sectional schematic of the waveguide used to investigate optical mode indices by employing eigenmode solver. (b) Optical mode at 1550 nm with effective index of 1.5693 for 0.6 μm wide and 0.5 μm thick optical waveguide. (c) Simulated effective mode indices for three different waveguide widths (W = 0.6 μm, 0.8 μm and 1.0 μm) of optical waveguide with a fixed height (H = 0.5 μm) for 1350 nm to 1560 nm optical range.

To calculate the group index, one needs to find the dispersion of the nonlinear waveguide. By using the eigenmode solver of finite element method based simulation tool, we investigated the effective mode indices of fundamental optical modes in the nonlinear optical waveguide considering the cross-section structure illustrated in Fig. 2(a). A SiO_2_ isolation layer with 1 μm thickness was used underneath the optical waveguide to prevent the evanescent coupling of the optical signal into the THz Si waveguide. We used the refractive index of aluminum nitride for the nonlinear waveguide in the eigenmode simulation. With a fixed waveguide height of 0.5 μm, we simulated the effective mode indices of the nonlinear waveguide for three different waveguide widths of 0.6 μm, 0.8 μm and 1.0 μm. Simulated dispersion characteristics of the nonlinear waveguide for the input infrared waves ranging from 1350 nm to 1560 nm is shown in Fig. 2(c). It is observed that effective mode indices of the fundamental optical mode in the optical waveguide decrease with the increase in wavelength. Also, effective index for the fundamental mode of a specific wavelength decreases with a decrease in width of the waveguide while we keep the height constant. Equation (2) suggests that group index and effective radius of the ring is inversely proportional. So we look forward to minimizing the radius of the ring resonator by maximizing the group index. Group index of the waveguide can be calculated from the dispersion curves shown in Fig. 2(c) by applying equation (3). We found the group indices as 2.43, 2.51 and 2.65 for three different waveguide widths of 1 μm, 0.8 μm and 0.6 μm, respectively with 0.5 μm height. It is evident that smaller waveguide dimension gives larger group index. However, for the width smaller than 0.6 μm, it becomes very hard to confine the infrared input waves in the waveguide. Thus 0.6 μm width and 0.5 μm height values were chosen for the nonlinear waveguide which resulted the fundamental mode of 1550 nm shown in Fig. 2(b). For the investigated device, the tunability resolution or *FSR* of the nonlinear ring is set to 0.05 THz. By applying the group index of 2.65 and *FSR* of 0.05 THz in equation (2), we calculate outer radius of the nonlinear optical ring resonator to be 360 μm.

**Figure 3:**
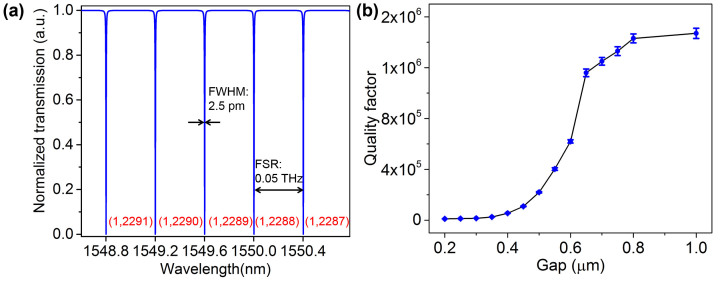
Transmission spectrum of optical ring resonator. (a) Transmission spectrum of the nonlinear optical micro-ring resonator with 360 μm radius, 0.6 μm width and 600 nm critical coupling gap. The Q factor and FSR are extracted to be 620,000 and 0.05 THz respectively. Numbers (red) at each absorption peak represent resonant mode number (radial, azimuthal) in the ring resonator for that wavelength. (b) The dependence of Q factor on the coupling gap of micro-ring resonator. The error bars represent the range for the infrared band of 1474 nm–1550 nm.

If the gap between the bus waveguide and the ring resonator is set to satisfy the critical coupling condition, the entire incident IR radiation couples to the ring resonator and the transmitted power in the bus waveguide drops to zero at resonant frequencies. This happens only when the coupled power is equal to the power loss in the ring, i.e., κ^2^ = 1 − *a*^2^, where κ^2^ is defined as the fraction of power coupling between the bus waveguide and the micro-ring resonator and *a* is the round trip amplitude transmission in the ring^23^,^24^. In order to achieve efficient difference frequency generation in 0.5–10 THz, critical coupling needs to be maintained over 1474 nm to 1550 nm infrared waves in the nonlinear ring resonator. For this infrared band, we simulate the amplitude transmission *a* in the nonlinear ring with radius of 360 μm to be 0.99. Critical coupling was achieved at a gap of 600 nm for which κ^2^ was found to be 0.02. Simulated transmission spectrum of the nonlinear optical ring resonator with 360 μm radius and 600 nm coupling gap is presented in Fig. 3(a). We also show the Q factor for different coupling gap sizes in Fig. 3(b). At critical coupling gap, optical Q factor is found to be 620,000 near 1550 nm. When the coupling gap is increased beyond this critical point, the ring is operated in a weakly coupled regime leading to improved Q factors around 1,500,000, approaching to the resonator's intrinsic quality factor.

Mode number for the resonant optical modes in the resonator can also be estimated by applying resonant wavelengths observed in transmission spectrum and their respective effective indices obtained from Fig. 2(c) in equation (1). For instance, at 1550 nm optical wave, we find the resonant mode number to be (1, 2288). Here ‘1' and ‘2288' represent radial and azimuthal mode number respectively. κ^2^ is defined as the fraction of power coupling between the bus waveguide and the micro-ring resonator. The waveguide power coupling coefficient κ^2^ and the propagation power loss coefficient κ_p_^2^ can be estimated from transmission spectrum of the ring resonator to be 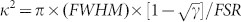 and 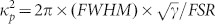, where *γ* is defined as the minimum power transmission in the through-port and FWHM is the full width at half maximum of the resonant peak^25^. To be compared with the losses in straight waveguides, which is often quoted in dB/cm, the propagation loss in a microring resonator can be expressed as 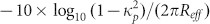, where 2*πR_eff_* is the perimeter of the microring resonator^25^. From the transmission spectrum of the critically coupled ring resonator over the infrared range of interest, we calculated the FWHM, FSR and *γ* to be 2.5 ± 0.1 pm, 0.4 ± 0.01 nm and 0.001 ± 0.0005. The estimated κ^2^ and κ_p_^2^ were 0.019 ± 0.0014, 0.0012 ± 0.0004, respectively, and the corresponding propagation loss was 0.023 ± 0.01 dB/cm. Using the value of minimum power transmission *γ*, we also found high extinction ratios of 30 ± 3 dB for the ring resonator over the infrared range of interest.

### Phase matching condition

Satisfying the phase matching condition (PMC) is the most challenging part of the design. Only if this condition is satisfied, the generated THz will co-propagate with the optical waves and show coherent amplification. Phase matching condition can be written as,
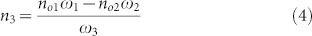
where *ω*_1_,*ω*_2_,*ω*_3_ are input pump, idler and the generated THz angular frequencies respectively and *n_o_*_1_,*n_o_*_2_,*n*_3_ are effective indices at pump, idler and THz frequencies respectively. Since we have already designed the nonlinear optical waveguide, the THz waveguide can now be engineered to meet the PMC. By applying the effective indices of infrared input waves obtained from Fig. 2(c) in equation (4), we found that the effective indices of THz modes have to be in the range of 2.6–2.7 for phase matching. Hence, THz waveguide must be designed in such a way that it supports and confines the THz modes with effective indices lying in this range.

It is well known that Si can be used to guide radiation in the near-infrared (NIR), and that high resistivity Si is relatively transparent in much of the THz. Thus, high resistivity Si waveguides were chosen to guide THz for its high refractive index and it can concentrate modes with much smaller than the size of the modes of ordinary optical fibers and can be efficiently coupled to nonlinear materials or polymers^26^,^27^. Loss tangent and attenuation coefficient of Si at any frequency can be calculated using the formulae tan*δ* = 1/(*ωε_Si_ε*_0_*ρ*) and 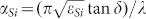 respectively, where *ρ* is resistivity of Si and *ε*_0_,*ε_Si_* are permittivity of free space and relative permittivity of Si respectively. For low impurity concentration *ε_Si_* is almost a real value, which is approximately equal to the high frequency relative permittivity. We estimate the loss tangent at 1 THz for 10 *k*Ω·*cm* high resistivity Si with *ε_Si_* = 11.67 to be 1.54 × 10^−5^ and the attenuation coefficient to be 0.55 m^−1^.

**Figure 4:**
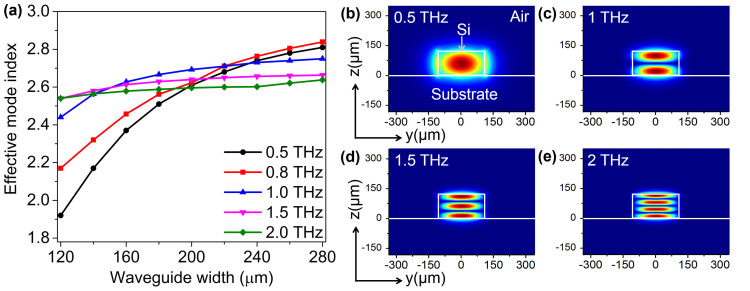
Engineering THz waveguide satisfying phase matching condition. (a) Simulated effective indices of the THz high resistivity Si waveguide with a fixed height of 120 μm by varying the waveguide width from 120 μm to 280 μm, showing the change in effective indices in THz waveguide for 0.5, 0.8, 1.0, 1.5 and 2.0 THz. Showing THz modal field profiles in the THz waveguide cross-section for (b) 0.5 THz (c) 1.0 THz (d) 1.5 THz and (e) 2.0 THz.

We investigated the dispersion of THz Si waveguides for different dimensions to find a suitable waveguide dimensions with required THz mode effective indices for phase matching. For THz waveguide with a fixed height of 120 μm, we simulated effective indices of five different THz frequencies by varying the waveguide width from 120 μm to 280 μm. It is observed that for a given mode, effective index increases with the increase in frequency if the waveguide dimensions are kept fixed. Also, for a given frequency, effective indices of higher order modes are always smaller compared to its lower order modes in a waveguide with fixed dimensions. Hence, modes of different orders were selected for the frequencies of interest in order to compare and find their effective indices in the range of 2.6–2.7^28^. The simulated results are shown in Fig. 4(a) for 0.5, 0.8, 1.0, 1.5 and 2.0 THz. It is clearly observed that for THz wave with smaller frequency and larger wavelength like 0.5 and 0.8 THz, effective mode indices increase rapidly with a small increase in waveguide width. But if one goes further to higher frequencies of THz waves, the change in effective indices becomes nearly insignificant for a small increase in waveguide width. This is due to the smaller wavelength for which it is easier to confine the wave and find a particular mode index in that large waveguide. Waveguide width of 200 μm was chosen for which effective mode indices were in the range 2.6–2.7 for all the five different THz waves presented in Fig. 4(a). THz modal profiles were simulated by eigenmode solver in the engineered THz waveguide with 200 μm width and 120 μm height to show the confinement of THz waves with required mode indices. Modal profiles satisfying phase matching condition in the THz waveguide are shown in Fig. 4(b), 4(c), 4(d) and 4(e) for 0.5, 1.0, 1.5 and 2.0 THz respectively.

### THz generation by DFG

A two-tier approach was adopted for THz generation simulations due to the large physical dimensions of the entire device for the 0.5–10 THz range. First, a smaller hybrid micro-ring resonator was designed and investigated by full 3D simulations using a commercially available FDTD tool and the results were compared with the ones obtained by 2D simulations. Once the accuracy of 2D simulations was confirmed, a larger hybrid micro-ring resonator for 0.5–10 THz range was designed and investigated.

The first design included a hybrid micro-ring resonator of 6 μm outer radius, with an optical ring resonator of 0.6 μm width and 0.5 μm thickness and input waveguides with the same dimensions. Underneath, a THz ring resonator and THz straight waveguides with the width of 3.5 μm and thickness of 2 μm were added to out-couple the generated THz radiation. Optical and THz structures were separated by a 1 μm thick SiO_2_ layer. For this small ring resonator, critical coupling was achieved at a gap of 500 nm. Second order nonlinear optical susceptibility χ^(2)^ was taken as 300 pm/V for the nonlinear optical ring resonator. Two optical beams at 1560 nm and 1350 nm were excited at two input straight optical waveguides, respectively, with electric field amplitude of 1 × 10^7^ V/m and bandwidth of 0.15 THz.

**Figure 5:**
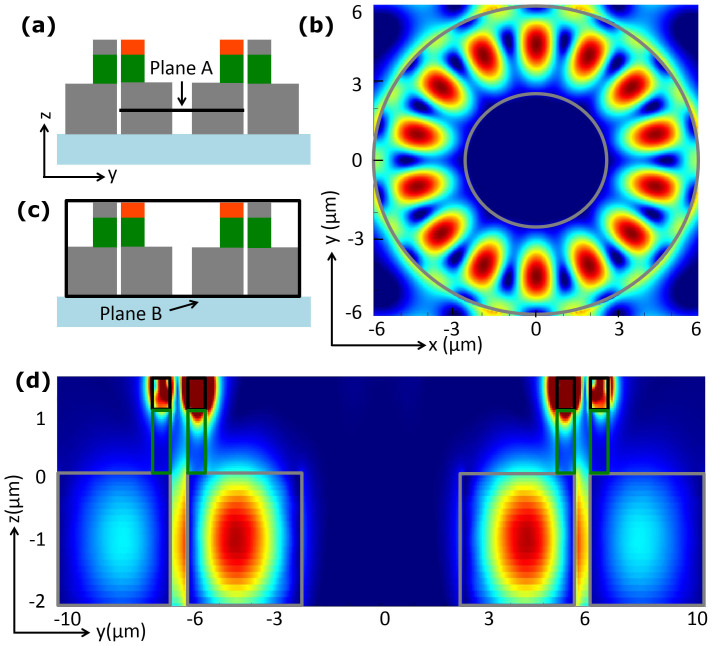
Electric field profiles obtained from 3D simulation of a hybrid optical and THz micro-ring resonator THz source with 6 μm radius. DFG is generated at 30 THz for two input optical waves at 1560 nm and 1350 nm. (a) Cross-section of the 3D THz source showing plane A in the THz ring resonator. (b) Showing 30 THz DFG field profile for plane A which is placed at XY plane in the THz ring resonator. It is clearly observed that the DFG THz is well confined in the ring with resonant mode. (c) Cross-section of the 3D THz source showing plane B. This plane is selected in order to observe the coupling of the THz generated in the nonlinear ring to THz ring resonator and THz straight waveguide as well. (d) Electric field profile on plane B where it is clearly observed that the DFG THz is coupling to the THz ring resonator placed underneath the nonlinear ring. It is also shown that THz is out-coupling from the ring resonator to the straight THz waveguide.

Electric field profiles at different planes from the 3D simulation were analyzed in order to fully understand how the proposed device works. First, electric field at the generated THz frequency was observed on plane A in the THz ring resonator placed underneath the nonlinear optical resonator as indicated in Fig. 5(a). The field profile on that plane is presented in Fig. 5(b) which clearly shows that the DFG THz wave is confined to the THz ring resonator with resonant mode. Then we observed electric field on another plane B as marked in Fig. 5(c). This plane is chosen in order to observe and prove if the THz wave generated in the nonlinear ring is coupled to the bottom THz ring resonator and also if it out-couples to the THz straight receiver waveguide from the ring resonator. The electric field presented in Fig. 5(d) evidently shows that the THz wave generated in the optical ring couples to the THz ring resonator and again from there out-couples to the THz receiver waveguide. Since we observe that 30 THz DFG radiation (10 μm wavelength) easily couples from the nonlinear top ring to the bottom THz waveguide ring through the 1 μm SiO_2_ layer, it is expected that in the large device, DFG generated THz radiation in 0.5–10 THz range (600 μm-30 μm wavelength) should more easily and efficiently couple to the bottom THz ring because of its larger wavelength.

**Figure 6:**
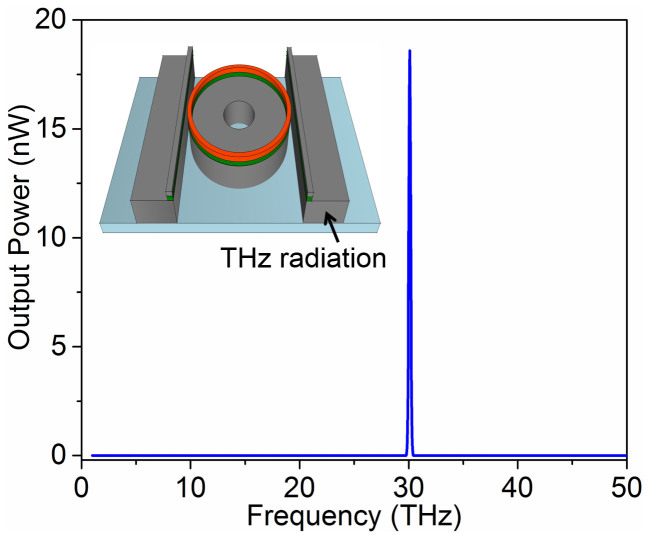
3D simulation result of a hybrid optical and THz micro-ring resonator THz source with 6 μm radius. Two input optical waves were excited at 1560 nm with resonant mode (1,35) and at 1350 nm with resonant mode (1,45). Sharp DFG peak at 30 THz is observed in the power spectrum obtained at the receiver THz straight waveguide. This result coincides exactly with the theoretical calculations involving DFG phenomenon.

Finally, the power spectrum at the output straight THz receiver waveguide was analyzed in order to observe the DFG peak. The output spectrum presented in Fig. 6 shows a clear DFG peak at 30 THz which is in good agreement with the theoretical calculations for DFG process^29^. The linewidth of the generated THz waves is found to be 0.3 THz which is two times the bandwidth we set for the input infrared wave.

Then, the 3D multi-layer structure was converted into a 2D simulation model for better computational efficiency. Since only one layer with ring resonator structure can be considered in 2D, we simulated only the top nonlinear optical ring resonator coupled with two input waveguides at two opposite sides. We selected the same χ^(2)^ value of 300 pm/V in the nonlinear ring and excited the same input waves at 1560 nm and 1350 nm with electric field amplitude of 1 × 10^7^ V/m and bandwidth of 0.15 THz. Simulating this geometry in 2D using the same FDTD tool resulted the exact same output characteristics proving the accuracy of the 2D simulations.

**Figure 7:**
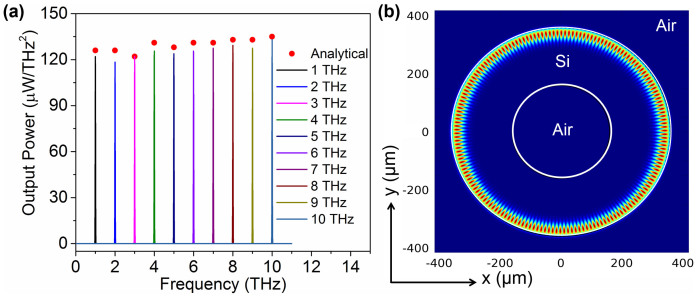
2D simulation result of the proposed THz source with 360 μm radius. (a) When one input beam was kept fixed at 1550 nm wavelength and the other input wavelength was varied and set to 1542 nm, 1534 nm, 1526 nm, 1519 nm, 1511 nm, 1503 nm, 1496 nm, 1488 nm, 1481 nm, and 1474 nm, sharp DFG peaks are observed near at 1 THz, 2 THz, 3 THz, 4 THz, 5 THz, 6 THz, 7 THz, 8 THz, 9 THz, and 10 THz, respectively, in power spectra at the receiver waveguide. This result coincides exactly with the theoretical calculations involving DFG phenomenon. Analytically estimated peak power at DFG THz frequencies are shown with circular dots. (b) Showing electric field distribution in the THz ring resonator for 5 THz. At this frequency, whispering gallery modes are observed causing the electric field to be confined at the outer boundary of the ring resonator.

In the second step, the previously described DFG emitter with 360 μm radius nonlinear ring coupled to two input optical bus waveguides was simulated in 2D using FDTD tool for output spectrum. The gap between the input bus waveguide and the ring resonator was kept at 600 nm for which critical coupling condition is satisfied. The same second order nonlinear optical susceptibility χ^(2)^ of 300 pm/V was used for the nonlinear ring resonator. Electric field amplitude and bandwidth of input waves were set to 1 × 10^7^ V/m and 0.015 THz, respectively. Resonant wavelengths obtained from the simulated transmission spectrum of the nonlinear ring resonator must be selected as inputs to generate a difference frequency in the ring. While the idler input wave was kept fixed at 1550 nm wavelength, the pump input wave was varied and set to 1542 nm, 1534 nm, 1526 nm, 1519 nm, 1511 nm, 1503 nm, 1496 nm, 1488 nm, 1481 nm and 1474 nm consequently to achieve difference frequency generation in the THz range of our interest. The simulated output power spectra for those different simulations are shown in Fig. 7(a). Sharp DFG peak is observed near at 1 THz, 2 THz, 3 THz, 4 THz, 5 THz, 6 THz, 7 THz, 8 THz, 9 THz, and 10 THz, respectively, in power spectra for those different input pump waves. The linewidth of the generated THz waves is found to be 0.03 THz which is two times the input pump bandwidth. The THz output power analytically estimated using equation (16) is also plotted along with the simulated results in Fig. 7(a) which shows a good agreement between the two. Generation of 5 THz radiation in the proposed device by DFG can be explained as follows. For the input, 1550 nm idler wave with resonant mode number (1, 2288) and 1511 nm pump wave with resonant mode number (1, 2388) are selected to satisfy the resonant condition in the nonlinear ring resonator. These two optical wave pulses are excited at the two ends of the input bus waveguides as it is shown in Fig. 1(a). Since they satisfy the resonance condition, the input waves make multiple round trips in high Q ring resonator cavity with enhanced optical intensity resulting in efficient DFG process. According to the DFG theory, these two waves incident upon a nonlinear material should produce the difference frequency field at 5 THz. Indeed, the simulated power spectrum in the ring resonator shown in Fig. 7(a) clearly presents a sharp DFG peak at 5 THz. In the proposed THz source, we expect this generated THz in the nonlinear ring to couple to the THz ring placed underneath as it is shown for a small scale 3D simulation in Fig. 5(d). Since both the optical and THz waveguides are engineered to satisfy the phase matching condition, DFG THz will travel in THz ring cavity with resonant mode and experience coherent amplification. Electric field distribution in the THz Si micro-ring resonator with whispering gallery resonant mode is presented in Fig. 7(b) for 5 THz DFG. THz straight waveguides placed at two opposite sides could out-couple the THz from the ring and guide it to any point of interest. Keeping the idler optical wave fixed at 1550 nm, the pump optical wave can be varied around this wavelength while satisfying the resonance condition of the ring to attain difference frequency output at the receiver end in the 0.5–10 THz range with tunability resolution of 0.05 THz.

### THz output power estimation

We estimated the THz output power of the proposed device analytically by employing the theory demonstrated in Methods section. Both optical beams were assumed to be at a power level of 0.5 W in the input bus waveguides. Some commercially available popular nonlinear materials and polymers such as aluminum nitride, potassium titanyl phosphate (KTP), GaSe and SEO100 polymer from Soluxra company with χ^(2)^ value of 5 pm/V, 27.4 pm/V, 108 pm/V and 500 pm/V respectively were used in the calculation. The THz output power of the device was estimated at 1 THz by considering the attenuation coefficient of 0.55 m^−1^ for high resistivity Si at 1 THz. For the analytical estimation, we used the values of round trip amplitude transmission and coupling coefficient to be 0.99 and 0.02 obtained from the ring resonator design. We calculated the THz output power at the receiver waveguide to be 2.2 μW, 66 μW, 1 mW and 27 mW, respectively, for the same nonlinear materials using equation (16). Considering the cross-sectional area of the THz receiver waveguide, the output intensity at 1 THz was estimated to be 92 W/m^2^, 2.75 kW/m^2^, 43 kW/m^2^ and 1.1 MW/m^2^, respectively for those materials.

## Discussion

Full 3D simulation of the proposed source device in broad spectral range poses computational challenges. The small scale emitter with 6 μm radius hybrid micro-ring resonator has a simulation volume of 25 *μm* × 25 *μm* × 10 *μm*. It takes approximately 60 hours on 16-core workstation meaning 960 CPU hours to complete the simulation. The estimated simulation volume for the proposed THz emitter is at least 1200 *μm* × 1200 *μm* × 300 *μm*. By considering only the volume ratio of simulation region, we estimate the 3D simulation of the proposed device to take approximately 66,355,200 CPU hours. Moreover, due to the high Q factor of the ring resonator, the simulation may take even longer for convergence. Therefore in this article we present 2D simulation results of the proposed THz source device along with 3D simulation results for a miniaturized version of the device in support of our claim. However, as stated above, the results obtained from a 2D and 3D simulation carried out for 6 μm radius hybrid micro-ring resonator are in a good agreement.

We used refractive index of aluminum nitride which is of 2.12 at 1550 nm to the optical waveguide in the simulation in order to investigate dispersion characteristics of the nonlinear waveguide and DFG simulation as well. Since refractive index of the nonlinear materials and polymers is typically around 2, there will be a slight change in the dimensions of the design if another nonlinear material or polymer is used instead of aluminum nitride. Nevertheless, the proposed hybrid ring resonator based THz source can be optimized easily for any other nonlinear materials by following the steps presented in details in Results section.

## Methods

### DFG in bulk nonlinear medium

Difference frequency generation (DFG) is a second order nonlinear optical process which generates an electromagnetic wave of frequency *ω*_3_ when two optical beams at slightly different frequencies *ω*_1_ and *ω*_2_ are incident upon a nonlinear material, such that the output frequency is the difference between the two input frequencies: *ω*_3_ = *ω*_1_ − *ω*_2_. Let us assume that a strong undepleted pump optical wave *E*^1^(*z*,*t*) and an idler optical wave of *E*^2^(*z*,*t*) with amplitudes *E_ω_*_1_ and *E_ω_*_2_ are propagating along with the generated THz field of *E*^3^(*z*,*t*) in *z* direction of the nonlinear medium^29^,
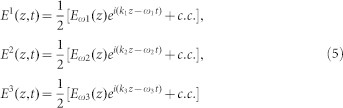
where, 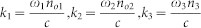 and *c.c.* stands for complex conjugate; *n_o_*_1_, *n_o_*_2_ and *n*_3_ are the effective mode indices for two optical beams and generated THz wave respectively. Second order nonlinear polarization is characterized by^30^,
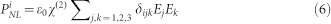
where, *δ*_123_ = 1,δ*_ijk_* is symmetric under all permutations of its indices and vanishes unless (*ijk*) are all distinct. Corresponding nonlinear polarization with the frequencies *ω*_1_, *ω*_2_ and *ω*_3_ are
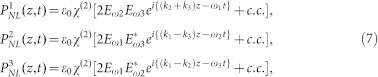


Now nonlinear polarization 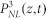 acts as a source for the propagation of THz field *E*^3^(*z*,*t*) in the *z* direction. So the governing wave equation is,
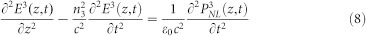


By substituting equation (5) and (7) into the wave equation (8), we obtain
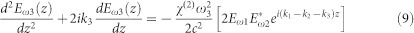


Invoking the slowly varying envelope approximation^29^, where the first term in equation (9) can be neglected since THz field amplitude *E_ω_*_3_ does not change appreciably for the propagation distance of a wavelength, the wave equation is reduced to
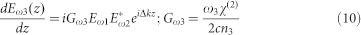
where, Δ*k* = *k*_1_ − *k*_2_ − *k*_3_ is the momentum mismatch. The amplitudes of the optical waves also vary slowly in the propagating direction and obey the similar wave equations,
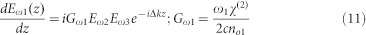

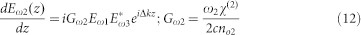


We solve these equations to find the THz field variation over distance *z* by assuming the phase matching condition holds, therefore, Δ*k* = 0 and pump wave field *E_ω_*_1_ is undepleted. The amplified idler wave field and the DFG THz field can be written as,
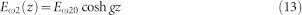

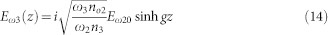
where 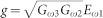 and *E_ω_*_20_ is the electric field of idler wave at *z* = 0.

### THz output power at the receiver waveguide

The THz field described in equation (14) is generated in the high Q optical ring resonator due to the interaction of two input infrared optical pump and idler waves carried in by the input waveguides placed at two sides of the nonlinear ring. We assume half circumference of the ring (*C*/2) to be the maximum travelled distance where two input waves interact with the nonlinear material resulting in THz generation without being changed due to out-coupling to the straight bus waveguides. This generated THz couples to the THz ring resonator where it is well confined with resonant mode. Then THz wave out-couples to the THz straight receiver waveguides placed at close proximity to the resonator. THz field at the receiver waveguide can be written as^31^,
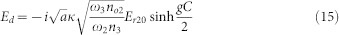
where *a* and *κ* are the total round trip amplitude transmission and power coupling coefficient in the ring resonator and *E_r_*_20_ is the initial electric field of idler wave in the ring resonator. If the area of the THz mode at the receiver waveguide is *A* and *n_eff_* is the effective mode index for the generated THz wave in the waveguide, then THz output power at the receiver waveguide can be estimated by the following formula,
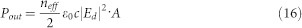


### Simulation modeling

We use a commercial finite element method simulation tool (COMSOL) to simulate both the nonlinear and THz waveguide dispersion. We attribute material dispersion of aluminum nitride to the nonlinear waveguide in mode calculations. For THz Si waveguide, we set a fixed refractive index of 3.42, since it remains same over the THz range of interest. We use another commercial finite difference time domain simulation tool (Lumerical) to run the 2D and 3D simulation of the proposed device to observe DFG.

## Author Contributions

N.P. conceived the idea. R.S., M.K. and N.P. discussed the theory and simulation method. R.S. designed, systematically investigated the source device and carried out the numerical simulations and analytical calculations. M.K. assisted R.S. with the 3D simulations. R.S. wrote the manuscript and prepared the figures. N.P. edited the manuscript. All authors discussed the results and commented on the manuscript. N.P. is the principal investigator of the project.

## Figures and Tables

**Table 1:** State-of-the-art THz Emitters

Emitter	Range(THz)	Power(W)	Temp.	Tunability	Size
Gun diode devices	0.01–0.2	10^0^–10^−4^	RT	Tunable	Medium
Resonant tunneling diodes (RTD)	0.1–1	10^−4^–10^−7^	RT	Not tunable	Medium
Backward wave oscillator (BWO)	0.3–1.3	0.05–10^−3^	RT	Tunable	Table Top
Free electron lasers	0.12–4.75	15 k–5 k	RT	Tunable	Bulky
Gas laser	0.9–3	10^−3^–.03	RT	Discrete lines	Table Top
Quantum cascade laser^32^	0.84–5	10^−4^–10^−1^	<169 K	Tunable	Small
